# Effects of Long-Term Storage on Radical Scavenging Properties and Phenolic Content of Kombucha from Black Tea

**DOI:** 10.3390/molecules26185474

**Published:** 2021-09-08

**Authors:** Chiara La Torre, Alessia Fazio, Paolino Caputo, Pierluigi Plastina, Maria Cristina Caroleo, Roberto Cannataro, Erika Cione

**Affiliations:** 1 Department of Pharmacy, Health and Nutritional Sciences, Department of Excellence 2018–2022, University of Calabria, Edificio Polifunzionale, 87036 Rende, Italy; latorre.chiara@libero.it (C.L.T.); pierluigi.plastina@unical.it (P.P.); mariacristinacaroleo@virgilio.it (M.C.C.); r.cannataro@gmail.com (R.C.); erika.cione@unical.it (E.C.); 2 Department of Chemistry and Chemical Technologies, University of Calabria, 87036 Rende, Italy; paolino.caputo@unical.it

**Keywords:** kombucha, black tea, long-term storage, antioxidant scavenging activity, total phenolic content

## Abstract

Kombucha is a fermented beverage. Its consumption has significantly increased during the last decades due to its perceived beneficial effects. For this reason, it has become a highly commercialized drink that is produced industrially. However, kombucha is still also a homemade beverage, and the parameters which, besides its organoleptic characteristics, define the duration of its potential beneficial properties over time, are poorly known. Therefore, this study aimed to determine the effect of 9-month storage at 4 °C with 30-day sampling on the pH, total phenolic, and flavonoid contents, free radical scavenging properties of kombucha fermented from black tea. Our results highlighted that, after four months, the phenolic content decreased significantly from the initial value of 234.1 ± 1.4 µg GAE mL^−1^ to 202.9 ± 2.1 µg GAE mL^−1^, as well its antioxidant capacity tested by two in vitro models, DPPH, and ABTS assays. Concomitantly, the pH value increased from 2.82 to 3.16. The novel findings of this pilot study revealed that kombucha from sugared black tea can be stored at refrigerator temperature for four months. After this period the antioxidant properties of kombucha are no longer retained.

## 1. Introduction

“Kombucha” is the name of a drink obtained by fermenting tea, mainly black or green, with the addition of sucrose, that acts as a substrate for fermentation, and a symbiotic culture of yeast and bacteria, known as “SCOBY” (Symbiotic Cultures of Bacteria and Yeast). The taste of this drink is slightly acidic and slightly carbonated, which makes it popular and pleasing to consumers [1]. Kombucha was first used in East Asia for its beneficial and curative effects only based on anecdotal evidences, since the Tsin dynasty began consuming it in Manchuria. It spread from China to Russia after World War I and then throughout Europe [1].

The fermentation is due to a symbiotic culture of acetic bacteria of the genus *Acetobacter* and *Gluconobacter* and different osmophilic yeast species, including genera such as *Saccharomycode*, *Schizosaccharomyces*, *Zygosaccharomyces*, *Brettanomyces/Dekkera*, *Candida*, *Torulospora*, *Koleckera*, and *Pichia* e *Mycoderma*. After fermentation, the kombucha tea is filtered through a cheesecloth and is consumed as a healthy drink. It can also be bottled for commercialization [2].

Almost forgotten for decades, kombucha became very popular again in the early 2000s, thanks to its sudden spread in Australia and in the United States. During the last decades, kombucha transitioned from a homemade fermented beverage to a soft drink produced on a large scale for commercial use [2,3]. Chemical analysis of kombucha beverage highlighted the presence of a variety of compounds, such as organic acids, mainly acetic, gluconic, and glucuronic acid, sugars (sucrose, glucose, and fructose), water-soluble vitamins (B1, B2, B6, B12, C), lipids, amino acids, biogenic amines, proteins, ethanol, minerals (manganese, iron, nickel, copper, zinc, plumb, cobalt, chromium, and cadmium), anions (fluoride, chloride, bromide, iodide, nitrate, phosphate, and sulphate), d-saccharic acid-1,4-lactone (DSL), carbon dioxide, and polyphenols [2,3]. Kombucha beverage is a source of bioactive components, such as glucuronic acid and polyphenols displaying antioxidant activity [4,5,6]. The low pH value of this beverage, especially owing to the presence of acetic acid in particular and a range of other organic acids, makes kombucha a drink with remarkable antimicrobial activity against a broad range of microorganisms [7,8,9,10] having also probiotic and symbiotic properties [11].

Many claimed beneficial effects of kombucha may be associated with its antioxidant activities, but when kombucha tea is stored at ordinary temperatures, the biofilm due to the presence of microorganisms continues to form, and might also affect the antioxidant activity. Epigallocatechin-3-gallate (EGCG) and epicatechin-3-gallate are converted into the corresponding epigallocatechin (EGC) and epicatechin (EC), the phenolic concentration in kombucha tea shows a linear increase during the fermentation time [11]. It is worth to note that the beneficial outcomes of the kombucha drink are mainly attributed to the activity of polyphenols, which in turn can act epigenetically [12,13].

Jayabalan et al. studied the effect of temperature (50–90 °C) on biochemical components and free radical scavenging properties of kombucha tea during a storage period of three months [14], concluding that heat treatment was not a suitable method for kombucha tea preservation. Therefore, it is of interest to elucidate the relationship between storage time and the changes of the antioxidant ability of kombucha. In fact, time, temperature, and light can significantly impact the quality and the biological activities of this beverage. To the best of our knowledge, no studies were carried out to determine the effects of storage times for more than three months and at low temperature. Therefore, this study aimed to evaluate the effects of the long-term storage at 4 °C on the pH, total phenolic and flavonoid contents, and free radical scavenging properties of kombucha during nine months with a sampling of 30 days, in order to evaluate the period during which these parameters are stable.

## 2. Results

### 2.1. Monitoring of the pH Values of Kombucha during the Stogare Period 

**Figure 1:**
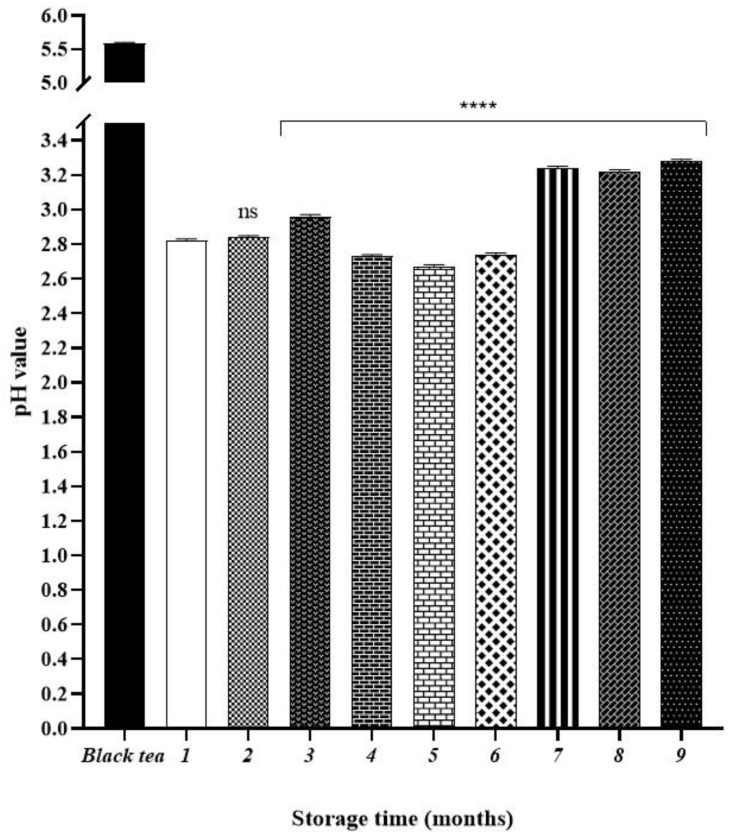
Values of pH determinate in black tea, kombucha tea after 30 days of fermentation and all the samples during storage. Black bar refers to the starting tea. White bar refers to the kombucha sample after 30 days of fermentation (control). Values represent the three-measure mean ± standard deviation. Asterisks on the bars indicate that mean values were statistically different from the control (**** *p* < 0.0001).

The pH values of black tea alone, as well as kombucha tea after one month of fermentation and all the samples over the storage are shown in Figure 1. It was observed that the pH value of sweetened black tea was 5.59, and it dropped to 2.82 in the kombucha beverage obtained after 30 days of fermentation (white bar) decreasing by about 2.77 units. This latter pH was used as the control for all the kombucha samples analyzed during nine months of storage. The value remained constant (2.84), till two months by decreasing significantly compared with the control (**** *p* < 0.0001) of about 0.2 units from months four to six. Then, the pH value of sample significantly increased up to 3.24, and it remained constant at these values at less than 0.05 units for the last three months (from months seven to nine).

### 2.2. Total Phenolic Content (TPC)

**Figure 2:**
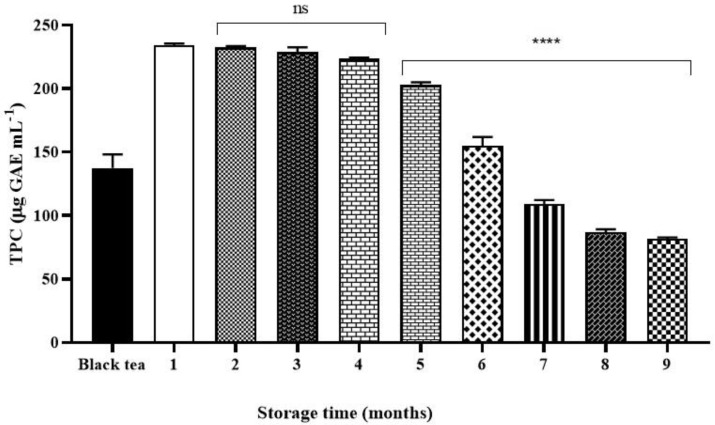
Total phenolic content (TPC) (µg GAE mL^−1^) of black tea, kombucha tea after 30 days of fermentation and all the samples during storage. Black bar refers to the starting tea. White bar refers to the kombucha sample after 30 days of fermentation (control). Values represent the three-measure mean ± standard deviation. Asterisks on the bars indicate that mean values were statistically different from the white bar that represents the control (**** *p* < 0.0001).

The total phenolic content (TPC) in all kombucha samples is shown in Figure 2. The results were expressed as µg equivalents of gallic acid (µg GAE) per mL of sample.

The results highlighted that the kombucha obtained after one month of fermentation, used as reference, showed the maximum TPC level which was 1.7 times higher (234.1 ± 1.4 µg GAE mL^−1^) than the value of black tea, (137.5 ± 10.7 µg GAE mL^−1^), respectively. In the following months (from months two to four), the TPC slowly decreased from 234.1 ± 1.4 to 223.5 ± 0.7 µg GAE mL^−1^ without significant statistic difference. On the contrary, it was decreasing in a time dependent manner, from months five to nine, significantly by about 13% (202.9 ± 2.1 µg GAE mL^−1^, **** *p* < 0.0001) at month five, and 34% at month nine (80.8 ± 5.4 µg GAE mL^−1^).

### 2.3. HPLC-DAD Analyses

**Figure 3:**
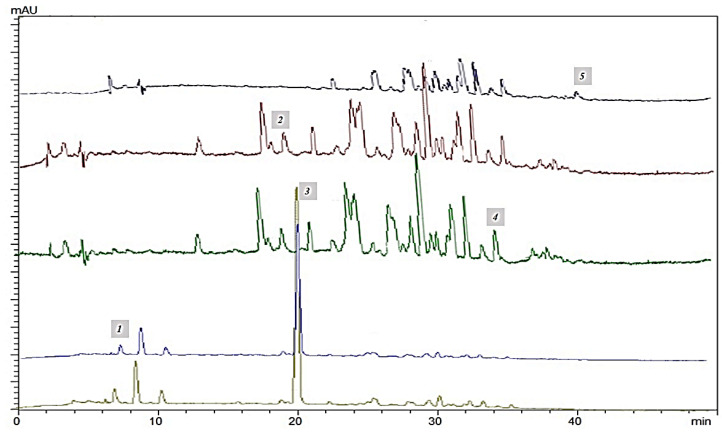
HPLC chromatogram of identified compounds in all the samples at the relative wavelengths to which they have been detected and quantified: 1. EGCG (λ = 280 nm); 2. Chlorogenic acid (λ = 327 nm); 3. Caffeine (λ = 273 nm); 4. Ferulic acid (λ = 325 nm); 5. Quercetin (λ = 365 nm).

**Figure 4:**
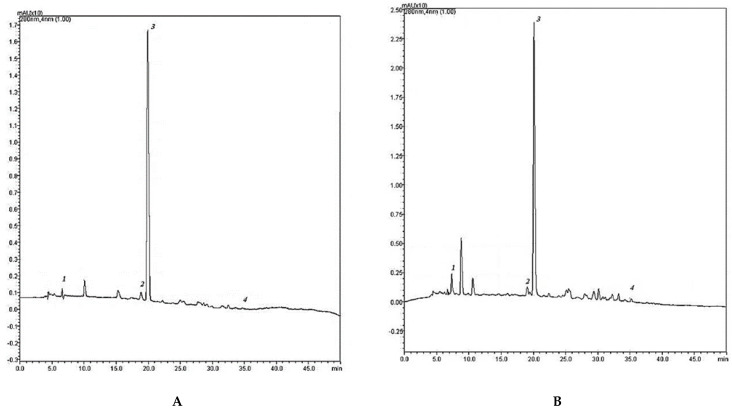

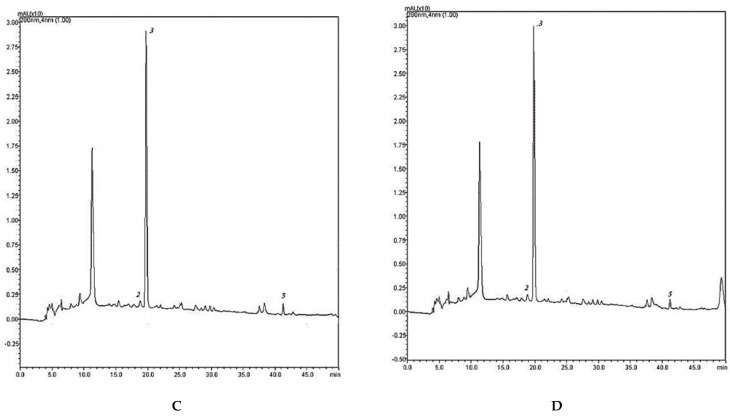
Chromatographic evolution at 280 nm including: (**A**) black tea; (**B**) kombucha after one month of fermentation; (**C**) kombucha tea sample at the fifth month of storage, when the epigallocatechin gallate content was not detected whereas quercetin, absent in black tea, was identifiable; (**D**) kombucha tea sample at the ninth month of storage.

Five compounds were identified and quantified by HPLC analyses in all samples (Figure 3). Chromatographic evolution at 280 nm is show in Figure 4A–D.

Caffeine was the main compound in all tea samples. Its initial value in black tea was 568.61 ± 0.84 µg mL^−1^ (Table 1), which underwent a reduction of 37.34% after fermentation, of 40.29% after one month and of 45.37% after two months. The minimum value was reached after four months (135.36 ± 1.63 µg mL^−1^) which corresponded to a reduction of the initial value of 76.19%. After six months the caffeine content increased (674.98 ± 0.49 µg mL^−1^), reaching its maximum value (702.93 ± 0.02 µg mL^−1^) after nine months. Chlorogenic acid was the only compound that maintained its content unchanged during fermentation and over time compared to the initial value in black tea (29.60 ± 0.01 µg mL^−1^). EGCG content in tea was 20.58 ± 0.32 and remained unchanged after 30 days of fermentation and in the next three months of storage, becoming undetectable at by the fourth month. A similar trend was displayed by ferulic acid, present in a smaller amount in black tea (3.15 ± 0.03 µg mL^−1^). Its content gradually decreased during fermentation in the next five months of storage, becoming undetectable at the sixth month. On the other hand, quercetin, absent in black tea, was identifiable only in the samples after four months from fermentation (23.07 ± 0.01 µg mL^−1^). Its content remained constant from months five to nine. The results are shown in Table 1.

### 2.4. Total Flavonoid Content (TFC)

**Figure 5:**
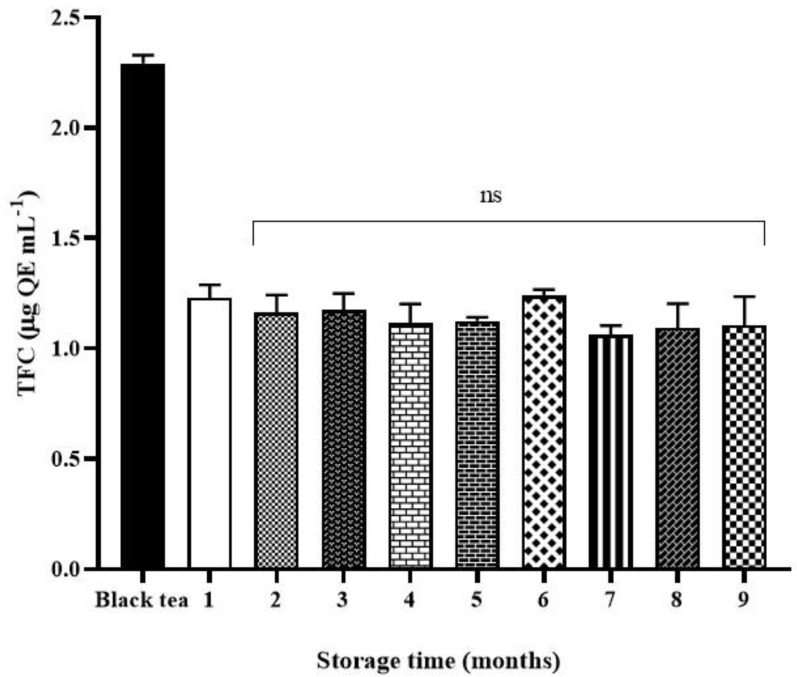
Total flavonoid content (TFC) (µg QE mL^−1^) of black tea, kombucha tea after 30 days of fermentation and all the samples over storage. Black bar refers to the starting tea. White bar refers to the kombucha sample after 30 days of fermentation (control).

Total flavonoid content (TFC) underwent a decrease during fermentation (Figure 5). The initial value of TFC in black tea (2.3 ± 0.2 QE mL^−1^) was reduced by 50% in kombucha tea (1.1 ± 0.3 µg QE mL^−1^) and it remained constant over time.

### 2.5. Determination of Antioxidant Activity

Scavenging abilities of black tea, kombucha tea after one month of fermentation and all the samples during the storage were monitored using two common in vitro models, DPPH and ABTS assays.

#### 2.5.1. DPPH Assay

**Figure 6:**
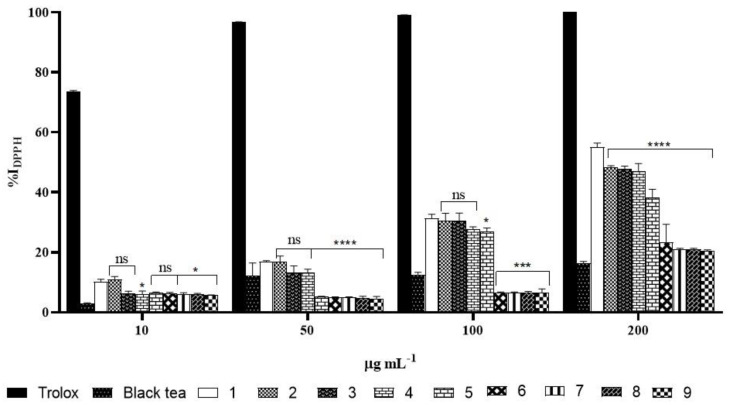
%I_DPPH_ of Trolox, black tea, kombucha tea after 30 days of fermentation and all the samples during storage. White bar refers to %I_DPPH_ of kombucha tea after 30 days of fermentation (control), the others refer to kombucha samples over nine months of storage. Asterisks on the bars indicate that mean values were statistically different from the control (* *p* < 0.05, *** *p* < 0.001, **** *p* < 0.0001).

Kombucha exhibited good antioxidant activity against DPPH radical during the storage at all tested concentrations when compared to black tea (Figure 6). The results of the DPPH radical assay were also expressed as the Trolox equivalent antioxidant capacity (TEAC) using Trolox as reference standard. The TEAC values are reported in Table 2. During fermentation, %I_DPPH_ of kombucha tea at the highest and lowest concentrations (55.1 ± 1.3 at 200 µL, and 10.8 ± 0.8 at 10 µL, respectively) increased by about 70% compared to that of black tea, which was 16.26 ± 0.7 (2.6 ± 0.1 µg TE mL^−1^) at 200 µL, and 3.1 ± 0.1 (0.8 ± 0.1 µg TE mL^−1^) at 10 µL. At the highest concentration, a 13% decrease was observed during the first three months of storage with respect to the control value, while it reduced by 30% after four months. At the fifth month it dropped drastically to a value of 23.2 ± 6.1% (3.0 ± 0.7 µg TE mL^−1^), corresponding to a reduction of 58% of the control value (**** *p* < 0.0001). The scavenging ability of kombucha declined to 18.9 ± 0.4% (2.9 ± 0.1 µg TE mL^−1^) after nine months.

#### 2.5.2. ABTS Assay

**Figure 7:**
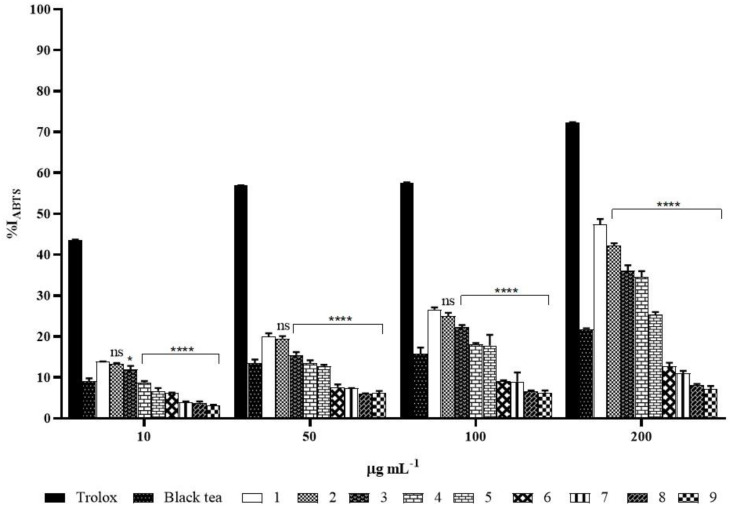
%I_ABTS_ of Trolox, black tea, kombucha tea after 30 days of fermentation and all the samples during storage. White bar (month 1) refers to %I_ABTS_ of kombucha tea after 30 days of fermentation (control), the others refer to kombucha samples over nine months of storage. Asterisks on the bars indicate that mean values were statistically different from the control (* *p* < 0.05, **** *p* < 0.0001).

The inhibition percentage of ABTS is reported in Figure 7 and the corresponding TEAC values against ABTS are reported in Table 3. Kombucha tea samples showed lower inhibitory abilities on ABTS•^+^ radical cation at the highest concentrations (100 and 200 µL) than those against DPPH radical. After one month of fermentation %I_ABTS_ of tea Kombucha at the highest concentrations (200 and 100 µL) increased by about 54% (47.4 ± 1.3, 9.1 ± 0.3 µg TE mL^−1^) and 40.6% (26.6 ± 0.5, 6.1 ± 0.1 µg TE mL^−1^) respectively, as compared to the black tea at the same concentrations, that is 21.8 ± 0.7 corresponding to 4.8 ± 0.1 µg TE mL^−1^ at 200 µL and 15.8 ± 1.5 or 3.4 ± 0.2 µg TE mL^−1^ at 100 µL. After one month of storage, the abilities of the samples at 200 (42.3 ± 0.5, 9.1 ± 0.3 µg TE mL^−1^) and 100 µL (25.0 ± 0.8, 5.5 ± 0.1 µg TE mL^−1^) were lowered by 11% and 6%, respectively, compared to the control. The antioxidant capacity of the control at the highest concentration underwent further but progressive reduction up to 27% in the first three months of storage, but it was reduced by 73% (%I_ABTS_ 12.7 ± 0.9, or 3.4 ± 0.2 µg TE mL^−1^) at the fifth month of storage and 85% (%I_ABTS_ 7.0 ± 0.4, or 1.6 ± 0.1 µg TE mL^−1^) at the ninth month.

## 3. Discussion

The consumption of kombucha has increased over the last decades due to its perceived beneficial effects. For this reason, it has become a highly commercialized drink, industrially produced but is still also a homemade beverage. To evaluate the effects of long-term storage of kombucha on radical scavenging properties and its phenolic content, we kept the tea samples at refrigerator temperature in the dark. Accordingly, to the literature, the pH values of black tea in the kombucha decreased after 30 days of fermentation due to the metabolic activity of tea fungus yeasts and acetic acid bacteria that produce mainly acetic acid [15].

In fact, the pH value of sweetened black tea was 5.59, and it dropped to 2.82 in the kombucha beverage obtained after 30 days. Then, the changes in the first five months of storage were less than 0.2 units, while at the sixth month, the pH value of sample significantly increased up to 3.24, and it remains constant at these values at less than 0.05 units for the last three months. This pH rise is most likely due to the subsequent use of acids by bacteria as a carbon source in the absence of sugar in the tea [16,17]. The final pH of kombucha samples (3.16), after nine months of storage, is still in the safe pH range of 2.5 to 4.2 for human consumption [18,19].

Total phenolic and flavonoid contents were also monitored after fermentation and during storage. The results highlighted that kombucha after one month of fermentation showed the highest total phenolic content level, which was 1.7 times higher than the value of black tea, as a consequence of the action of microbial enzymes from bacteria and yeasts in an acidic environment, which hydrolyzes complex tea polyphenols into smaller molecular weight phenolic compounds causing an increase in polyphenol concentration [19]. Total phenolic content is reduced after four months of storage. A similar trend was seen for the total flavonoid content but earlier than the total phenolic content.

In contrast to Ning et al. [20], HPLC analysis pointed out that chlorogenic acid maintained its content unchanged either during fermentation and over time compared to the initial value in black tea. On the other hand, all the other monitored phenolics dropped at the fifth month. This agrees with the literature in which the strategy to prolong phenolic content is studied [20]. Conversely, total flavonoid content was constantly lower in black tea, most likely due to the microbial activity of the SCOBY during fermentation.

The results of DPPH inhibition properties of kombucha tea directly depend on the tea constituents and the components produced during fermentation time (30 days). The decrease of antioxidant capacity during storage was most likely related to microbial transformation of the compounds responsible for the maximum scavenging ability into less potential scavenging structures. On the other hands, the inhibition percentage of ABTS assay showed lower inhibitory abilities in respect to ABTS•^+^ radical action.

The antioxidant activities of our tested samples likely depend on the composition and the chemical nature of phenolic compounds [21,22]. Then, during the storage, changes in the composition of antioxidant compounds of kombucha tea might result from the formation of certain compounds as in the case of quercetin in our results, thus leading to a lower antioxidant activity [22].

It is important to mention here, that during the COVID-19 pandemic of 2019–2020 the consumption of fermented food, especially beverages, increased in several countries [17,23,24]. In particular, the consumption of industrial kefir and kombucha increased [15,25] and the latter was reported, in the magazine Forbes, as the drink of 2020 [26]. Although, as source of bioactive components that could be beneficial for human health, there is no evidence about systematic human trials being done using kombucha tea [27] and some toxicity related to kombucha consumption has been reported so far when kept in a ceramic pot for six months or in lead-glazed earthenware at refrigerator temperature [28,29].

## 4. Materials and Methods

### 4.1. Standards and Chemicals

**Figure 8:**
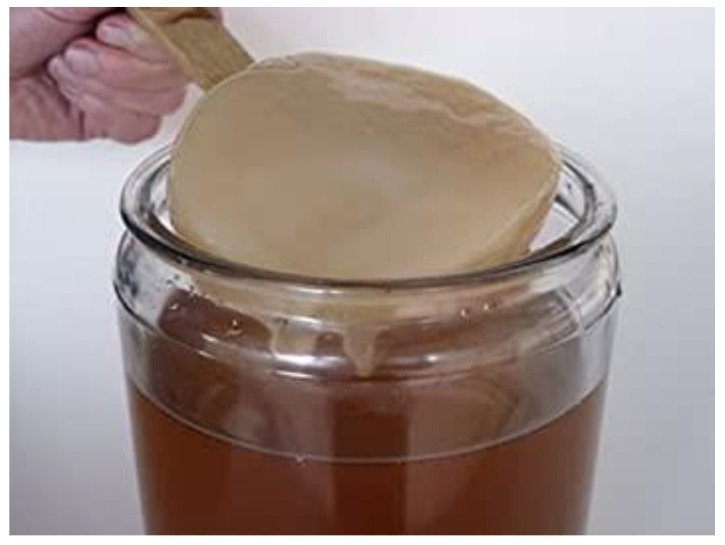
Kombucha starter, the “SCOBY”.

The kombucha starter (Figure 8) was obtained from Kefiralia (Burumart Commerce S.L, Arrasate, Spain) and was maintained in sugared black tea. Dimethylsulphoxide (DMSO), absolute ethanol and methanol, formic acid, and acetonitrile HPLC-grade were purchased from Carlo Erba (Milan, Italy), Folin-Ciocâlteu reagents, sodium carbonate, DPPH, ABTS, potassium persulfate (K_2_S_2_O_8_), aluminium chloride (AlCl_3_), potassium acetate, chloroform, and ethyl acetate were purchased from Sigma Aldrich (Milan, Italy).

### 4.2. Preparation of Kombucha Tea and Fermentation Conditions

Black tea (3 g) was immersed into 1 L of boiling water and infused for about 15 min. Then it was filtered through a sterile sieve. This was repeated for three times and 1 L of each preparation was kept into sterilized glass jars. Commercial sucrose (7%) was then added to the hot drink and, after cooling to room temperature, the infusion was inoculated with a commercial kombucha SCOBY (150 g) size (15 × 2 × 10 cm). The jars were covered with a clean cloth. The fermentation was carried out in the dark at 25 ± 2 °C for 30 days and, at the end of this time, the kombucha tea samples were filtered through a cheesecloth and transferred to three amber jars.

### 4.3. Storage Condition

The jars containing kombucha tea were placed in a refrigerator (T = 4 °C) for nine months. Sampling was performed every 30 days by taking an aliquot (100 mL) which was analyzed. The tea fermented for one month was used as the control for the kombucha tea samples stored at 4 °C for longer times—up to nine months. pH values, content of total polyphenol compounds, qualitative and quantitative profile of the main tea polyphenols, content of total flavonoids, and free radical scavenging activities of each sample were determined.

### 4.4. pH Values of Tea Kombucha during Storage

The pH values of all the kombucha tea samples were measured using an electronic pH meter (Hanna Instruments, George Washington Hwy, Smithfield, RI, USA) calibrated at pH 4.0 and 7.0.

### 4.5. Total Phenolic Content (TPC)

The total polyphenolic content (TPC) compounds in the tea samples were quantified by the Folin-Ciocâlteu colorimetric method as previously described [30], with appropriate modifications. The fermented tea sample (0.1 mL) was transferred in an amber glass vial and was added by 2 mL of distilled water, 0.5 mL of the Folin-Ciocâlteu reagent (diluted 1:10 with distilled water), and 0.4 mL of a 7.5% sodium carbonate solution (Na_2_CO_3_), up to a final volume of 3 mL. The mixture was shaken under constant magnetic stirring for 30 min, at room temperature in the dark. The absorbance was measured at 760 nm using a spectrophotometer Jasco UV-550. Three analyses were carried out for each sample. Gallic acid was used as the standard in order to plot the calibration curve. For the linearity study, an eight-point calibration curve was constructed using different concentrations of gallic acid stock solutions (range 0.5–0.01 mg mL^−1^). A linear correlation was found between absorbance of the blue complex at 760 nm and concentration of gallic acid in the range 0.5–0.01 mg mL^−1^ (y = 3.6607x − 0.0036). The coefficient (R^2^) obtained from the linear regression was 0.9998, indicating an excellent linear correlation between the data. The total phenolic content (TPC) was expressed as µg equivalents of gallic acid (µg GAE) per mL of kombucha.

### 4.6. HPLC-DAD Analyses

Five compounds in kombucha tea samples were identified and quantified by reversed-phase high performance liquid chromatography coupled with diode array detector (HPLC-DAD) [31]. The samples were filtered through a membrane filter (0.45 µm) into HPLC vials and analyzed as such. An aliquot (10 µL) of each sample was injected into a Shimadzu (Kyoto, Japan) HPLC system equipped with a diode array detector (SPD-M10Avp). The chromatographic separation was performed on a Mediterranea SEA C-18 column (4.6 mm i.d. × 25 cm, 5 μm). The mobile phase was a 0.1% formic acid (A) and acetonitrile (B) mixture. The gradient used was the following: 0 min, 10% B; 20 min, 22% B; 40 min, 40% B; 45 min, 10% B, 51 min, 10% B. The flow rate and column temperature were maintained as 0.6 mL min^−1^ and at room temperature, respectively. Detection was made at the absorption maxima of the pure standard compounds: caffeine was detected at 273 nm, EGCG at 280 nm, ferulic acid at 325 nm, chlorogenic acid at 327 nm, and quercetin at 365 nm, and identification was made by comparison of the retention times and characteristic UV-Vis spectra of pure standard compounds used as references. Individual components were analyzed quantitatively by the external standard method. The calibration curves for standards (caffeine, EGCG, ferulic acid, chlorogenic acid, and quercetin) were prepared with six appropriate concentrations. The limit of detection (LOD) and the limit of quantification (LOQ) for each standard were calculated as follows: LOD = 3(S_y_/S) and LOQ = 10(S_y_/S), where S_y_ is the standard deviation of the response of the curve and S is the slope of the calibration curve.

### 4.7. Total Flavonoid Content (TFC)

Total flavonoid content (TFC) was determined by a colorimetric method as described previously [32]. Briefly, 0.30 mL of the sample solution were diluted with 1.68 mL of distilled water. Then, 0.9 mL of MeOH, 0.06 mL of a 10% AlCl_3_ solution, and 0.06 mL of 1 M solution of potassium acetate were added to the solution. The mixture was allowed to stand for 30 min at room temperature, under constant magnetic stirring, in the dark, and then the absorbance was measured against the blank at 420 nm using a spectrophotometer Jasco UV-550. Three analyses were carried out for each sample. Quercetin was used as the standard in order to plot an eight-point calibration curve. The linearity range of calibration curve was 10–0.001 µg mL^−1^ (y = 0.084x − 0.0019). The coefficient (R^2^) obtained from the linear regression was 0.9984, indicating a good linear correlation between the data. The results were expressed as µg of quercetin equivalents (µg QE) per mL of kombucha [31].

### 4.8. Radical Scavenging Activity

Two different in vitro assays, DPPH and ABTS, were used to evaluate the changes over time in free-radical scavenging abilities of all kombucha tea samples.

#### 4.8.1. DPPH Assay

The scavenging activity on DPPH radical was determined by the colorimetric method previously described [25] with slight modification. To different volumes of each sample (10, 50, 100, 200 µL), 0.1 mL of DPPH solution (1 mM) and 2.8 mL of MeOH were added. After an incubation time of 30 min, under magnetic stirring, at room temperature and in the dark, the reduction of DPPH free radical was measured by reading the absorbance at 517 nm using a spectrophotometer Jasco UV-550. The experiments were carried out against a blank (3 mL of MeOH) and a control (2.9 mL of MeOH, 0.1 mL DPPH solution). Each sample was tested in triplicate. The antioxidant activity was given as a percentage of free radical inhibition (%I_DPPH_), according to the formula:

%I_DPPH_ = [(absorbance of the control − absorbance of the sample)/absorbance of the control)] × 100.

The results were expressed also as µg of Trolox equivalents per mL of tea samples (µg TE mL^−1^).

#### 4.8.2. ABTS Assay

ABTS•^+^ radical cation is well soluble in both aqueous and organic solvents, so this method can be extensively used to determine antioxidant activity for both hydrophilic and lipophilic compounds [33]. This radical cation was formed by a reaction between 7 mM ABTS solution and 2.45 mM potassium persulfate (K_2_S_2_O_8_), and then allowing the mixture to stand for 16 h in darkness at room temperature. It remains stable for the following 48 h, and is characterized by an intense green/blue color. ABTS•^+^ solution was diluted in methanol until the absorbance reached the value of 0.70 ± 0.02 at 734 nm. Different volumes of each tea broth sample (10, 50, 100, 200 µL) were mixed with ABTS solution (3 mL), and the absorbance was recorded at 734 nm after 10 min of incubation at room temperature in the dark, against a blank and a control. Each sample was tested in triplicate. The radical scavenging activity was given as percentage of ABTS•^+^ radical inhibition (%I_ABTS_), according to the following formula:

%I_ABTS_ = [(absorbance of the control − absorbance of the sample)/absorbance of the control)] × 100.

The results were expressed as µg of Trolox equivalents per mL of tea kombucha samples (µg TE mL^−1^).

### 4.9. Statistical Analysis

Each tea broth from three different preparations, was analyzed in triplicate and, the results were expressed as mean ± standard deviation (SD). One-way ANOVA method and a Holm-Sidak comparison method via GraphPad Prism 8 were used. The significance was established at *p* values < 0.05 (*), *p* < 0.01 (**), *p* < 0.001 (***), and *p* < 0.0001 (****).

## 5. Conclusions

The polyphenol content of kombucha during long-term storage decreases significantly from the fifth month on and becomes one-third of the initial value after nine months. Therefore, a period of up to four months ensures the preservation of polyphenols of kombucha tea and their antioxidant activities. The results of this pilot study highlighted that the “shelf life” of kombucha stored at refrigerator temperature could be no longer than four months, as only during this period the preservation of polyphenol content and its antioxidant activities are ensured.

## Figures and Tables

**Table 1:** Content of HPLC identified compounds, expressed as µg mL^−1^.

Samples	Caffeine	Chlorogenic Acid	EGCG	Ferulic Acid	Quercetin
Black tea	568.61 ± 0.84	29.70 ± 0.01	20.58 ± 0.32	3.15 ± 0.03	nd ^a^
Fermented Kombucha	356.25 ± 7.17	29.60 ± 0.03	20.24 ± 0.53	2.97 ± 0.90	nd
1st storage month	339.50 ± 4.15	29.51 ± 0.06	19.90 ± 0.12	1.21 ± 0.01	nd
2nd storage month	310.63 ± 0.97	29.56 ± 0.01	19.96 ± 0.07	1.30 ± 0.20	nd
3rd storage month	159.20 ± 0.55	29.24 ± 0.38	19.92 ± 0	0.82 ± 0.36	nd
4th storage month	135.36 ± 1.63	29.41 ± 0.41	nd	0.75 ± 0	nd
5th storage month	162.60 ± 0.26	29.64 ± 0.91	nd	0.41 ± 0.04	23.07 ± 0.01
6th storage month	674.98 ± 0.49	29.72 ± 0.01	nd	nd	23.16 ± 0.59
7th storage month	672.27 ± 0.07	29.58 ± 0.03	nd	nd	23.23 ± 0.08
8th storage month	702.91 ± 0.71	29.72 ± 0.01	nd	nd	23.03 ± 0.07
9th storage month	702.93 ± 0.02	29.60 ± 0.01	nd	nd	23.04 ± 0.03

^a^ Not-detected.

**Table 2:** Trolox equivalent antioxidant capacity (TEAC) values (µg TE mL^−1^) of different samples against DPPH.

Samples	Concentrations			
	10 µL	50 µL	100 µL	200 µL
Black tea	0.8 ± 0.1	2.0 ± 0.6	2.1 ± 0.1	2.6 ± 0.1
Fermented Kombucha	1.2 ± 0.1	2.1 ± 0.1	3.0 ± 0.2	7.4 ± 0.2
1st storage month	1.3 ± 0.4	2.1 ± 0.3	4.3 ± 0.2	6.3 ± 0.1
2nd storage month	0.7 ± 0.1	1.5 ± 0.3	4.0 ± 0.2	6.4 ± 0.3
3rd storage month	0.6 ± 1.0	1.7 ± 0.2	3.6 ± 0.1	6.3 ± 0.2
4th storage month	0.4 ± 0.1	0.5 ± 0.1	3.4 ± 0.1	5.1 ± 0.4
5th storage month	0.5 ± 0.1	0.5 ± 0.1	0.8 ± 0.1	3.0 ± 0.7
6th storage month	0.6 ± 0.1	0.7 ± 0.1	0.8 ± 0.1	2.8 ± 0.3
7th storage month	0.7 ± 0.1	1.3 ± 0.1	2.0 ± 0.2	2.7 ± 0.1
8th storage month	0.6 ± 0.1	0.9 ± 0.1	1.0 ± 0.6	3.0 ± 0.3
9th storage month	0.6 ± 0.1	0.9 ± 0.1	1.2 ± 0.1	2.9 ± 0.1

**Table 3:** Trolox equivalent antioxidant capacity (TEAC) values (µg TE mL^−1^) of different samples against ABTS.

Samples	Concentrations			
	10 µL	50 µL	100 µL	200 µL
Black tea	1.8 ± 0.2	3.0 ± 0.2	3.4 ± 0.2	4.8 ± 0.1
Fermented Kombucha	2.8 ± 0.1	4.5 ± 0.1	6.1 ± 0.1	9.6 ± 0.3
1st storage month	2.8 ± 0.1	4.4 ± 0.1	5.5 ± 0.1	9.1 ± 0.3
2nd storage month	2.6 ± 0.1	3.3 ± 0.3	5.1 ± 0.2	7.9 ± 0.2
3rd storage month	1.9 ± 0.1	2.8 ± 0.2	4.1 ± 0.7	7.5 ± 0.3
4th storage month	1.4 ± 0.2	2.8 ± 0.1	4.0 ± 0.5	5.7 ± 0.1
5th storage month	1.2 ± 0.5	1.6 ± 0.2	2.3 ± 0.5	3.4 ± 0.2
6th storage month	0.7 ± 0.1	1.6 ± 0.1	1.9 ± 0.1	3.3 ± 0.1
7th storage month	0.5 ± 0.1	1.3 ± 0.1	1.4 ± 0.1	1.6 ± 0.1
8th storage month	0.4 ± 0.1	1.1 ± 0.1	1.3 ± 0.1	1.5 ± 0.3
9th storage month	0	0	1.3 ± 0.2	1.6 ± 0.1

## Data Availability

The data presented in this study are available on request from the corresponding author.

## Sample Availability

Samples of the compounds are available from the authors.

## Abbreviations

EtOH	Ethanol
MeOH	methanol
DMSO	dimethylsulfoxide
TPC	total phenolic content
TFC	total flavonoid content
DPPH	2,2’-diphenyl-1-picrylhydrazyl
ABTS	2,2’-azino-bis(3-ethylbenzothiazolin-6-sulfonic)
Trolox	6-hydroxy-2,5,7,8-tetramethylcroman-2-carboxylic acid
HPLC	High Performance Liquid Chromatography
QE	quercetin equivalents
GAE	gallic acid equivalents
EGCG	epigallocatechin-3-gallate
ECG	epicatechin-3-gallate
EGC	epigallocatechin
EC	epicatechin
